# Magnetocapacitance in La_0.7_Sr_0.3_MnO_3_/Pb(Zr_0.2_Ti_0.8_)O_3_/La_0.7_Sr_0.3_MnO_3_ multiferroic heterostructures

**DOI:** 10.1038/s41598-017-06487-3

**Published:** 2017-07-26

**Authors:** Luminita M. Hrib, Lucian Pintilie, Marin Alexe

**Affiliations:** 1 National Institute of Materials Physics, Atomistilor 405bis, Magurele, Romania; 2 University of Warwick, Department of Physics, CV4 7AL Coventry, UK

## Abstract

Measurements of the magnetocapacitance effect in epitaxial La_0.7_Sr_0.3_MnO_3_/Pb(Zr_0.2_Ti_0.8_)O_3_/La_0.7_Sr_0.3_MnO_3_ heterostructures have been performed using a quasi-static method. Through capacitance-voltage measurements carried out under variable magnetic field it has been found that the magneto-capacitance depends on the orientation of the ferroelectric polarization. The value of magneto-capacitance can be as high as 1% in the voltage range near the ferroelectric coercive field. This has been attributed to a variation of the apparent built-in voltage of the PZT-LSMO Schottky barriers on applied magnetic field.

## Introduction

Ferroelectric-ferromagnetic multiferroic thin films heterostructures have been intensively studied due to their potential applications in spintronic devices, magnetoelectric sensors, transducers and energy harvesting devices^1^. The most appealing property of these composite heterostructures is the coupling between electric and magnetic properties, known also as magnetoelectric (ME) coupling, which may offer the control of ferroelectric polarization via magnetic field and, vice-versa, the spins via electric field. ME coupling in this type of heterostructure is only interfacial and requires a detailed study of the electronic phenomena at the interface between the ferromagnetic and ferroelectric materials. One of the most convenient methods to evaluate experimentally ME coupling is by measuring the electrical capacitance in a magnetic field, the magnetocapacitance (MC)^2, 3^. Although this method is simple the interpretation of the MC results can be challenging as artifacts such as the Maxwell-Wagner effect can lead to magnetocapacitance without true magnetoelectric coupling^4, 5^.

Most of the MC studies on multiferroic systems are simply performed by slowly sweeping the magnetic field while measuring capacitance at a constant DC and AC applied field. This type of measurement reveals basically the static coupling between the ordering parameters, i.e. polarization and magnetization, which in most systems is strain-mediated^6–8^. Alternatively, the MC coupling is determined by measuring the capacitance with temperature and by crossing one of the transition points where one of the order parameters is vanishing usually resulting in a discontinuity in temperature dependence of the relevant parameters^9^.

Here we study the MC coupling in an epitaxial La_0.7_Sr_0.3_MnO_3_/Pb(Zr_0.2_Ti_0.8_)O_3_/La_0.7_Sr_0.3_MnO_3_ (LSMO-PZT-LSMO) heterostructure caused by the switching of ferroelectric polarization. Capacitance-voltage (CV) measurements were performed on epitaxial LSMO-PZT-LSMO heterostructures. In order to gain more insight on the origin of the observed MC effect a model was developed and used to simulate the experimental data. This suggests an interface-related coupling responsible for the observed MC effect. More precisely, the apparent built-in potential of the LSMO-PZT Schottky barrier is magnetic field dependent.

## Results and Discussion

PZT and LSMO offer a series of advantages that are: i) similar perovskite structure with rather small lattice misfit with the STO substrate and with each other, which allows preparation of high quality epitaxial structures with sharp interfaces between PZT and LSMO; ii) PZT is a ferroelectric material with very high polarization value approaching 100 µC/cm^2^ in fully strained films grown on STO^10^; iii) the proven interaction between PZT and LSMO which goes beyond a simple electrostatic interaction at the interface^11, 12^.

Arguably most of the work related to magneto-electric coupling has addressed the static ME coupling, i.e. measured relevant parameters before and after ferroelectric or ferromagnetic switching or at a timescale each state can be considered a quasi-steady-state^13^, and only very few are measuring in a time scale relevant to ferroelectric switching^14^. We are measuring here capacitance-voltage (C-V) characteristics which offer the possibility to obtain information on the ME coupling while the ferroelectric polarization switches from one orientation to another.

**Figure 1:**
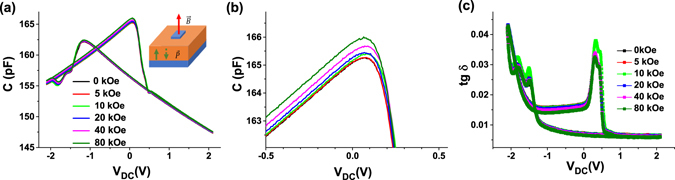
(**a**) Capacitance-voltage characteristics at different magnetic field acquired at 75 K and 1 kHz on a LSMO-PZT-LSMO sample with 50 nm PZT film thickness; (**b**) a zoom-in of (**a**) in the peak region associated with the polarization switching. The inset in (**a**) shows the measurement geometry; (**c**) Dielectric loss-voltage characteristics acquired at different magnetic fields.

Figure 1 shows typical C-V and dielectric loss (tgδ)-voltage characteristics of LSMO-PZT-LSMO capacitors acquired at different applied magnetic fields. CV characteristics show the typical butterfly shape with peaks at voltages corresponding to ferroelectric switching. After switching the capacitance has a linear voltage dependence, suggesting the existence of an interface capacitance. The magnetic field has a markedly influence on the capacitance, especially before and at the (positive) peak associated to ferroelectric switching.

In order to understand the mechanism of this ME coupling we model our system as two back-to-back Schottky diodes, as has been previously proposed for a generic metal-ferroelectric-metal heterostructure^15^. It is obvious that the above structure is quite asymmetric, including a rather high imprint which is rather stable. Given the fact that the structure is with the top and bottom LSMO electrodes obtained in the same deposition conditions, the asymmetry of the Schottky LSMO-PZT barrier is not unusual. Different LSMO-PZT interfaces are due to different processing (the bottom LSMO was deposited on the TiO_2_-terminated SrTiO_3_ monocrystalline while the top LSMO was deposited on top of the PZT film) and of different thermal treatments (the bottom LSMO was subjected to high temperature for a longer period of time) different densities of electronic states can exist at the interfaces. Previous studies show that even in the case of epitaxial interfaces asymmetries of the interfaces can be induced due to different chemical terminations of the subsequent layers^16^.

Despite the existing imprint and asymmetry, the ME coupling here can be analyzed defining two parameters: magnetocapacitance *MC* and magnetolosses *Mtgδ*:

1 $$MC=(\frac{C(H)}{C(H=0)}-1)100$$

2 $$Mtg\delta =(\frac{tg\delta (H)}{tg\delta (H=0)}-1)100$$

**Figure 2:**
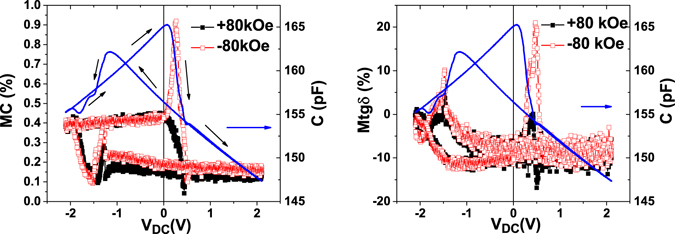
Magnetocapacitance (*MC)* and magnetolosses (*Mtgδ*) as function of the bias voltage for a LSMO-PZT-LSMO with PZT (50 nm). The acquired original data is superimposed (blue line). The black arrows indicate how the DC voltage is applied.

Both *MC* and *Mtgδ* parameters along with the pristine data are plotted in Fig. 2.

**Figure 3:**
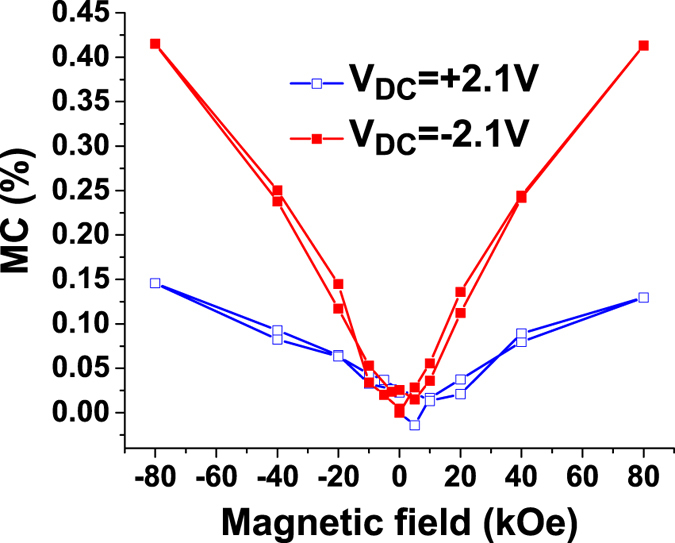
*MC* values for the data shown in Fig. 2 as a function of the magnetic field at large applied bias with no ferroelectric switching.

It can be observed that both *MC* and *Mtgδ* depend strongly on the applied DC voltage and magnetic field. A relative large asymmetry in the MC is observed. The *MC* after polarization switching is almost constant and is larger in for negative bias (~0.41%) than for positive (~0.2%) and is non-zero even at zero applied bias. This indicates that the observed magneto-capacitive effect is not necessarily due to a strain-mediated electromechanical coupling. This asymmetry of MC exists at all applied magnetic fields, as shown in Fig. 3. It is also obvious that exactly at the coercive voltage, when polarization switches from one direction to the other, *MC* shows a marked peak. This peak is also asymmetric in magnetic field, as can be easily inferred from Fig. 2.

A *MC* value as high as 1% is obtained at the coercive voltage suggesting a possible dynamic coupling between magnetization and polarization, potentially generated by an influence of the magnetic field on the movement of the dipoles. A similar process has been previously reported^17^ to occur in a Fe-doped BaTiO_3_ single crystal. An interface-induced magnetocapacitance as large as 15% and the decrease of the loss tangent was observed after applying a 90 kOe magnetic field to the sample. It was assumed that the magnetic field affects the density and the moment of the relaxing dipoles which further lead to the modification of the capacitance values. However in the case of the heterostructure studied in this paper, apart from the asymmetry with magnetic field, the MC at voltages corresponding to ferroelectric switching has very different values at zero magnetic field. This fact implies that during ferroelectric switching the magneto-capacitance effect is strongly affected by other phenomena that are not directly related to the magnetic field, as the remanent magnetization in LSMO or its actual domain pattern.

In order to understand the above results and to rule out any potential influence of Maxwell-Wagner or similar effects we have to perform an in-depth analysis of the results. The capacitance experimental data shown above is inferred by the LCR bridge from measured impedance values considering a simple parallel RC circuit. However, the measured impedance comprises many contributions originating from the electrodes, ferroelectric volume and interfaces. The heterostructure analyzed in this study contains a strong magnetoresistive material, LSMO, Schottky barriers at the interface between LSMO and PZT, and the ferroelectric PZT film. All these have non-linear behavior either in applied electric field, magnetic field, or both.

**Figure 4:**
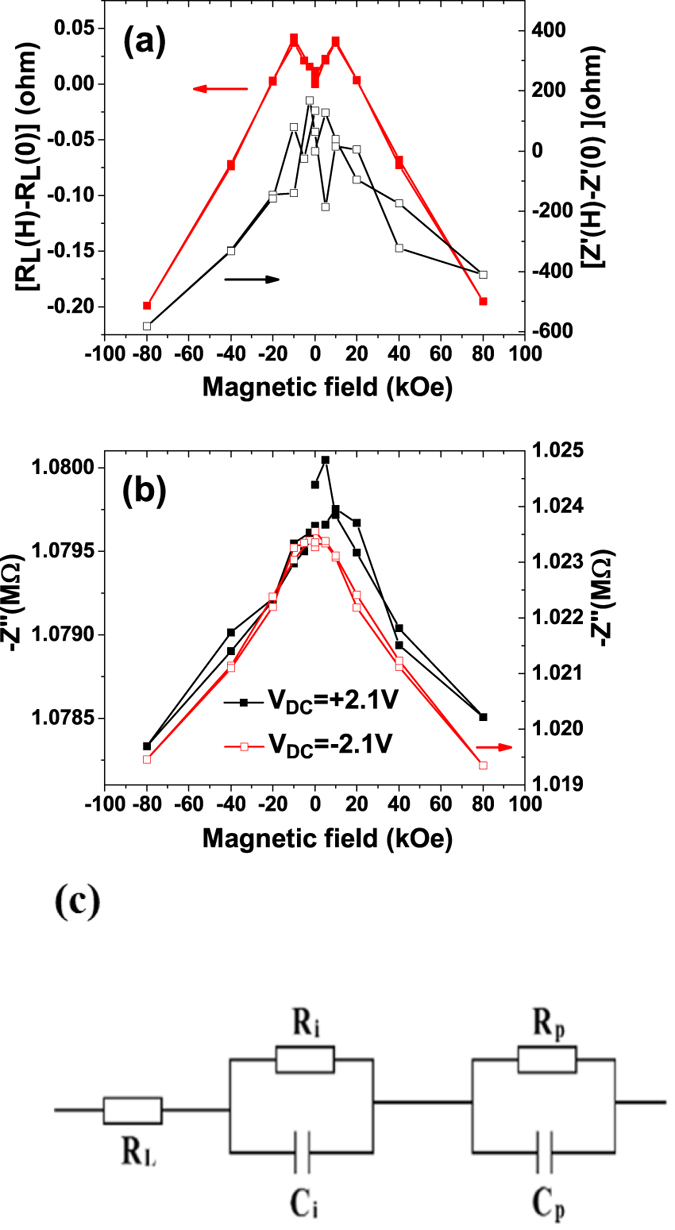
(**a**) Experimental DC magnetoresistance measured for a 20 nm thick epitaxial LSMO layer deposited on (100)-oriented STO using the same parameters as for the heterostructure (red full squares) and *Z*_*t*_′*(H)* − *Z*_*t*_′*(0)* for the LSMO-PZT-LSMO heterostructure for V_DC_ = +2.1 V; (**b**) Magnetic field dependence of imaginary part of the impedance *Z*″ at large applied DC voltages; (**c**) The equivalent circuit used for the simulation of the experimental impedance.

We first analyze the potential effect of the intrinsic magnetoresistance (*MR*) of the LSMO electrodes on the above MC data. In order to do this, we model the LSMO/PZT/LSMO structure by an equivalent circuit (Fig. 4(c)). The LSMO layer has a finite resistance *R*_*L*_ and the Schottky diodes are modeled as a series resistance (representing the semiconductor bulk resistance) connected in series with a parallel circuit comprising a voltage dependent capacitance and a conductance^18^. The aim is to calculate back the impedance of the structure from the capacitance data and to compare the variation of this impedance with the experimental values of MR of a single LSMO layer. The final equivalent circuit comprises of a resistance (*R*_*L*_) associated to the LSMO electrodes and two parallel RC circuits: one associated to the interfaces with the electrodes (*C*_*i*_ and *R*_*i*_) and one attributed to the ferroelectric volume (C_p_ and R_p_). It is assumed that the only magnetic field dependent component is *R*_*L*_ while *C*_*p*_, *R*_*p*_, *C*_*i*_ and *R*_*i*_ do not depend on the magnetic field.

Real *Z*_*t*_′ and imaginary *Z*_*t*_″ parts of the electrical impedance of the equivalent circuit are given by:

3 $${Z}_{t}^{\prime} ={R}_{L}+\frac{{R}_{i}}{1+{(\omega {C}_{i}{R}_{i})}^{2}}+\frac{{R}_{p}}{1+{(\omega {C}_{p}{R}_{p})}^{2}}$$

4 $${Z}_{t}^{\prime\prime} =\frac{\omega {C}_{i}{{R}_{i}}^{2}}{1+{(\omega {C}_{i}{R}_{i})}^{2}}+\frac{\omega {C}_{p}{{R}_{p}}^{2}}{1+{(\omega {C}_{p}{R}_{p})}^{2}}$$

and the total magneto-capacitance and magneto-losses (see S3 from Supplementary):

5 $$MC=(\frac{1+t{{g}_{t}}^{2}(0)}{1+t{{g}_{t}}^{2}(H)}-1)100$$

6 $$Mtg\delta =m({R}_{L}(H)-{R}_{L}(0))100$$

According to this model the magneto-capacitance is due to *Z*_*t*_′ that has a magnetic field dependence due to *R*_*L*_ while *Z*_*t*_″ should be independent on the magnetic field. Moreover, *Z*_*t*_′*(H)* − *Z*_*t*_′*(0)* = *R*_*L*_ *(H) *−* R*_*L*_ *(0)*. The experimental results of *R*_*L*_ *(H)* − *R*_*L*_ *(0)* and *Z*_*t*_′*(H)* − *Z*_*t*_′*(0)* as a function of the magnetic field are represented in Fig. 4(a). The experimental values for *R*_*L*_ were obtained on a 20 nm thick LSMO thin film prepared in the same conditions as those used for the LSMO-PZT-LSMO heterostructure.

It can be observed from Fig. 4(a) that, although the value of the LSMO resistance and of *Z*′ share a similar magnetic field dependence, the DC MR is four orders of magnitude lower than *Z*_*t*_′*(H)* − *Z*_*t*_′*(0)*. This result and the fact that *Z*″ has a magnetic field dependence (see Fig. 4(b)) of about 0.41%, similar to that observed for MC indicates that the magnetoresistance of the LSMO layers has a negligible contribution to the overall magneto-capacitance of the LSMO-PZT-LSMO heterostructure.

**Figure 5:**
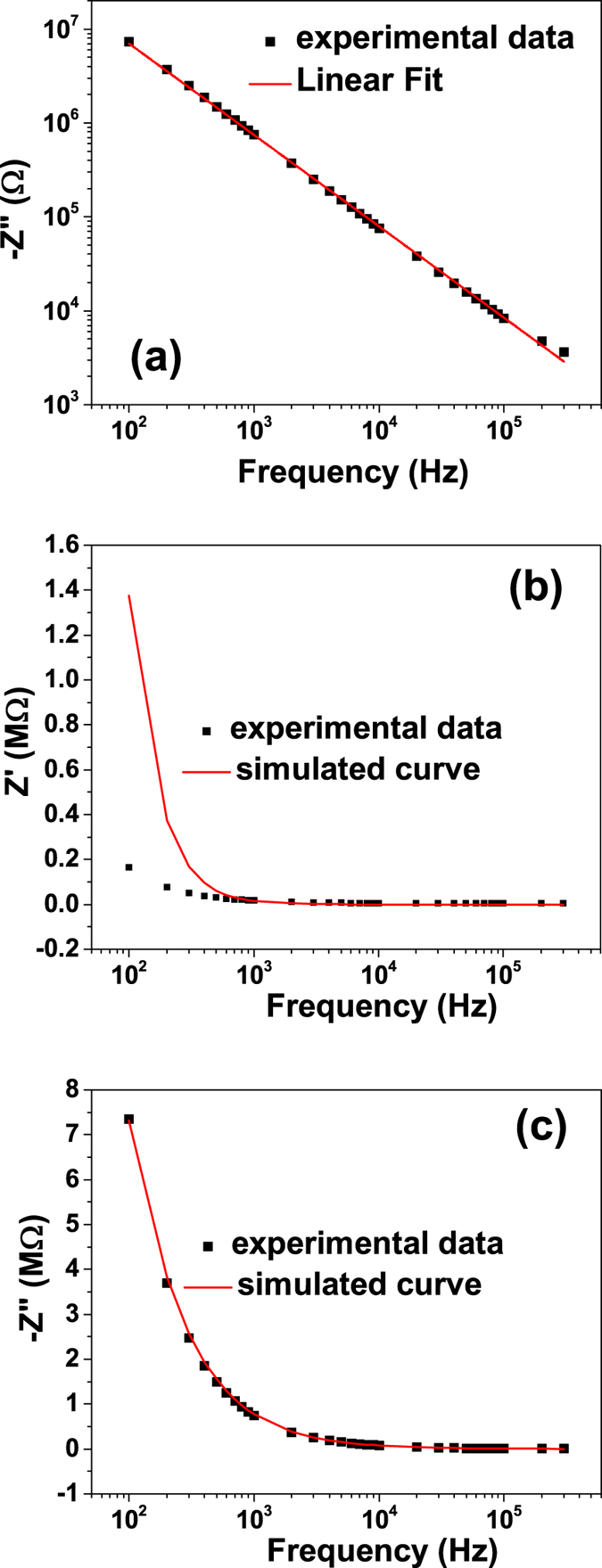
(**a**) log-log plot of frequency dependence of *Z*″; Simulated curve using the equivalent circuit from Fig. 4(c) along with the experimental data obtained for (**b**) *Z*′ and (**c**) *Z*″ with V_DC_ = 0 V and no external magnetic field.

The frequency dependence of the experimental zero field *Z*″ (H = 0 kOe and V_DC_ = 0 V) is presented in Fig. 5(a). This dependence is linear, with a slope of −0.97, very close to −1, indicating that *Z*″ is related to a common capacitive relaxation. This result suggests that the magnetic field variation of the −*Z*″ is solely due to the magnetocapacitance. However from this result it is not clear if the capacitance affected by the magnetic field is associated to the ferroelectric layer or to the interface capacitance, or both.

The capacitance of the ferroelectric layer *C*_*p*_, of the interface *C*_*i*_ and the other values of the equivalent circuit components (*R*_*i*_ and *R*_*p*_) can be obtained by fitting the dielectric dispersion curve of the heterostructure measured at zero field (Fig. 5(b) and (c)). Using the value of *R*_*L*_ at zero field, *R*_*p*_ = 12 Mohm, *R*_*i*_ = 300 Mohm, *C*_*p*_ = 370 pF and *C*_*i*_ = 450 pF in equation (3) and (4) the theoretical curve fits well the experimental results for *Z*″ and *Z*′ only above 1 kHz. The large difference between simulation and experimental results observed below 1 kHz for *Z*′ could be due to the presence of trapping centers that respond to the ac signal, affecting in this way the resistive part of the impedance. Nevertheless, since the measurements are performed at 1 kHz, we may assume that these effects are not affecting the discussion above.

At 1 kHz, the frequency at which the CV measurements were performed, the term (ωCR)^2^ ≫ 1 and *Z*″ can be approximated to:

7 $$Z^{\prime\prime} =\frac{1}{\omega }(\frac{1}{{C}_{i}}+\frac{1}{{C}_{p}})=\frac{1}{\omega C}$$

From this relation it results that at 1 kHz the imaginary part of the complex impedance of the heterostructure is equal to the equivalent impedance of a serial connection between two capacitors: one associated to the ferroelectric volume and one to the interfaces. Assuming that the capacitance of the epitaxial PZT layer does not depend on the magnetic field, the only reasonable explanation for the observed H-dependence of *Z*″ is a magnetic field dependence of the interface capacitance *C*_*i*_, which actually would explain also the global MC effect of the LSMO/PZT/LSMO stack presented in Fig. 2.

The values of the interface capacitance *C*_*i*_ as a function of the magnetic field are:

8 $$\frac{1}{{C}_{i}(H)}=\frac{1}{C(H)}-\frac{1}{C(0)}+\frac{1}{{C}_{i}(0)}$$

**Figure 6:**
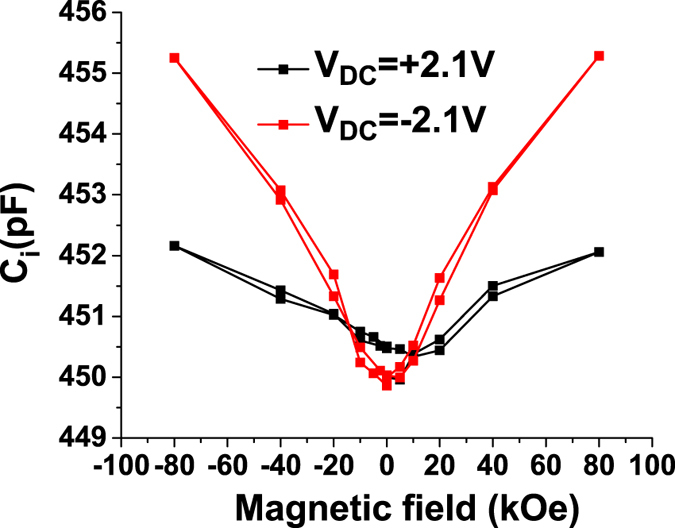
Magnetic field dependence of the interface capacitance *C*_*i*_ determined at high applied bias.

The *C*_*i*_ values as a function of the magnetic field for V_DC_ = + 2.1 V and V_DC_ = −2.1 V are presented in Fig. 6.

It is worth noting that a magneto-capacitance effect was observed in other types of materials that form Schottky barriers, such as Au/GaAs:Si^19^. Here the magnetic field shifts in the built-in potential by increasing the binding energy of the shallow Si impurity atoms in the presence of the DC magnetic field. Following a similar reasoning we can assume that the MC effect in the present of a metal-ferroelectric Schottky barrier case is due to influence of the magnetic field on the built-in potential. We have shown that the ferroelectric polarization alters the band bending at the metal ferroelectric interface and the built-in potential is in fact an apparent built-in potential that takes into account the influence of ferroelectric polarization. In this case the capacitance can be written as^20^:

9 $$C=\sqrt{\frac{q{\varepsilon }_{o}{\varepsilon }_{st}n}{2(V+V{}_{bi}^{\prime} )}}$$

where the apparent potential *V*_*bi*_′ is:

10 $${V}_{bi}^{\prime} ={V}_{bi}\pm \frac{P}{{\varepsilon }_{o}{\varepsilon }_{st}}\delta $$

where *C* is the specific capacitance, *q* is the electron charge, *ε*_*o*_ is the permittivity of vacuum, *ε*_*st*_ is the static dielectric constant, *n* is the density of free charge, *V*_*bi*_ is the built-in potential in the absence of polarization, *P* is the ferroelectric polarization, and *δ* is the distance between the polarization surface charge and the physical metal-ferroelectric interface. The +/− sign corresponds to the downwards/upwards orientation of the ferroelectric polarizations with respect to the substrate. According to equation (9), the capacitance values depend on the ferroelectric polarization orientation, especially at low voltage. However as the external DC voltage increases the polarization term has a smaller impact on the capacitance values.

**Figure 7:**
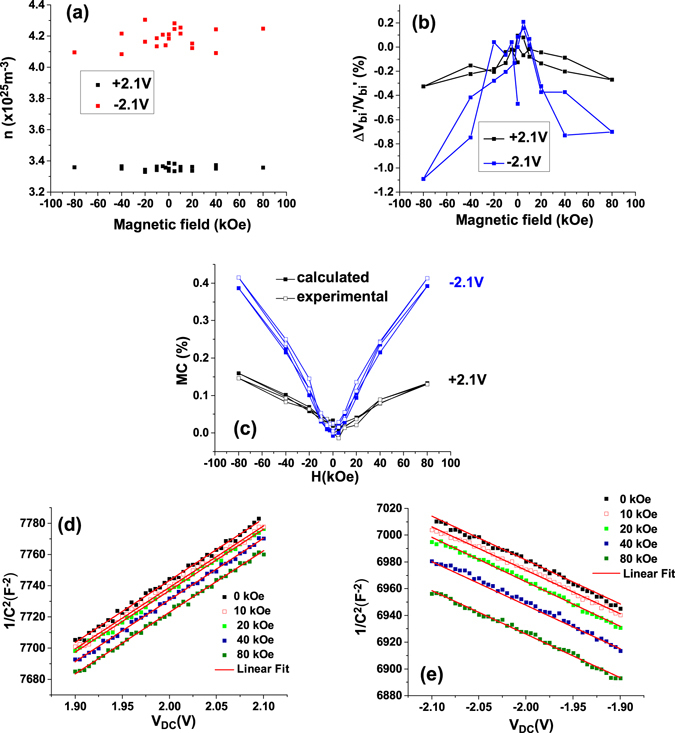
(**a**) Magnetic field dependence of the free carrier concentration and (**b**) of the apparent built-in potential *V*_*bi*_′; (**c**) comparison between the experimental (open symbols) and calculated (full symbols) *MC* values. The lines shown on (**b**) and (**c**) are only as guides to the eye; (**d**) and (**e**) *1/C* ^*2*^ vs *V*_*DC*_ and the linear fits.

By representing *1/C* ^*2*^ as a function of *V*_*DC*_ (Fig. 7(d) and (e)) for applied bias voltages at which the ferroelectric polarization is saturated and for different values of the magnetic field, the carrier concentration *n* can be estimated from the slope and the apparent built-in voltage *V*_*bi*_′ from the intercept. As shown in Fig. 7(a), the carrier concentration is (3.8 ± 0.5)10^25^ m^−3^ and, as expected, is not affected by the magnetic field. The small difference for the two polarization orientations of the polarization should actually be related to the asymmetry of the electrode interfaces, leading to different densities of interface states for holes and electrons at the two interfaces.

The apparent built-in potential depends on the ferroelectric polarization orientation as predicted by equation (10), as well as on magnetic field. A maximum variation of ~1% is obtained when the magnetic field reaches −80 kOe (Fig. 7(b)). The *n* and *V*_*bi*_′ values were used in (9) to simulate the magneto-capacitance values (solid points). The static dielectric constant *ε*_*st*_ was determined from the C-V measurements performed on PZT samples with different thickness (see S2 from Supplementary). Figure 7(c) shows the raw data and the simulated values of magneto-capacitance and, as can be observed, there is a good agreement between the calculated and experimental values for both orientations of the ferroelectric polarization.

The presented data along with the above discussion reveals a novel magneto-electric coupling at the LSMO-PZT interface. This type of ME coupling is genuinely electronic and is mainly a result of a magnetic field dependence of the built-in voltage of the Schottky barrier developed between PZT and LSMO. This built-in voltage is the result of the band alignment at the interface to accommodate the difference in electron chemical potential (Fermi level) in both materials. Ferroelectric polarization influences massively both materials at the interface. It affects directly the barrier formation and the band bending within the semiconducting PZT as well as the electronic properties of LSMO at the interface. Ferroelectric polarization not only it induces a carrier modulation in LSMO, or a simple change of the electron population of the Mn-3d bands^21^, but also induces a structural distortion of the MO_6_ octahedra responsible for the magnetic properties^22^. These polarization-induced structural and electronic changes of the LSMO interface layer have important effects on the electronic barrier formation. The built-in voltage of the PZT-LSMO Schottky barrier, which is a pure electronic parameter, experiences now an indirect magnetic field dependency generating a significant magneto-capacitance effect. As expected the effect increases when a certain instability is induced. By driving the DC voltage in the range of coercive voltage the polarization switching process contributes dynamically to the increase of the ME coupling.

## Conclusion

The magneto-capacitance of LSMO-PZT-LSMO heterostructures was analyzed at low temperature by performing capacitance-voltage measurements. It is found a rather constant magneto-capacitance value of about 0.41% at applied voltages outside the ferroelectric switching range. The effective value of magneto-capacitance depends on the ferroelectric polarization orientation and increases significantly at coercive voltages when the ferroelectric polarization switches from one direction to the other. We have proven that the magneto-capacitance effect is not stemming into Maxwell-Wagner relaxation mechanism or the innate magnetoresistance of the LSMO electrodes, but it is rather due to the magnetic field variation of the built-in voltage of the PZT-LSMO Schottky barrier. The origin of this magneto-electric effect is the ferroelectric polarization which is naturally affected by the band alignment at the interface, orbital occupancy and structure of the interfacial LSMO. The present work suggests a new route of increasing the magneto-electric effect by using dynamic effects induced by ferroelectric switching under magnetic fields in ferroelectric capacitor devices with ferromagnetic electrodes.

## Methods

The present study is performed on symmetric ferroelectric-ferromagnetic La_0.7_Sr_0.3_MnO_3_(20 nm)/Pb(Zr_0.2_Ti_0.8_)O_3_(50 nm)/La_0.7_Sr_0.3_MnO_3_(20 nm) thin film heterostructures grown on (100) -oriented SrTiO_3_ (STO) single crystals using pulsed laser deposition method.

The capacitance of 0.013 mm^2^ devices was measured using a Precision LCR Meter Agilent E4980A with ac excitation voltage of 0.2 V amplitude at 1 kHz frequency. C-V characteristics were obtained by scanning the applied DC bias voltage in a typical hysteretic sequence: 0 V →  + *V*_*max*_ → 0 V → −*V*_*max*_ → 0 V with sweeping speed of about 25 mV/sec. A magnetic field was applied perpendicular to the sample surface in a similar hysteretic way: 0 kOe →  + 80 kOe → 0 kOe → −80 kOe → 0 kOe. All measurements were performed at (75 ± 0.01) K in a Physical Property Measurement System (PPMS) Model 6000. Special care was taken to obtain the most reliable data in order to rule out any potential artefacts which would affect the measurements. It was observed that the CV characteristic of pristine device is markedly different than the subsequent measurements (see Supplementary Fig. S1). Therefore, mock CV characteristics were run 3–4 times on each device before final data acquisition. Moreover, since the expected effects are very low, we took special care to achieve good stability. The measurements were performed after 5 h from the moment the temperature of the system became stable and zero field capacitance has a drift of maximum 0.05% within 30 min. Finally, three CV characteristics were acquired for each value of the magnetic field. The CV characteristics analyzed were obtained by averaging the second and the third C-V curve. The DC magnetoresistance of LSMO layer grown in the same conditions as for the final heterostructures was measured using a 200 µm long Hall bar.

## Electronic supplementary material

Supplementary Information
