# Supplementary material for: Magnetocapacitance in La0.7Sr0.3MnO3/Pb(Zr0.2Ti0.8)O3/La0.7Sr0.3MnO3 multiferroic heterostructures
Source: Sci Rep. 2017 Jul 26;7:6563. doi: 10.1038/s41598-017-06487-3 (PMC5529449; doi:10.1038/s41598-017-06487-3)
Supplement: Supplementary file 1 — Supplementary Information [file 41598_2017_6487_MOESM1_ESM.pdf]

Supplementary Information

Magnetocapacitance in $La_{0.7}Sr_{0.3}MnO_3/Pb(Zr_{0.2}Ti_{0.8})O_3/La_{0.7}Sr_{0.3}MnO_3$ multiferroic heterostructures

Luminita M. Hrib[1*], Lucian Pintilie[1], Marin Alexe[2]

[1] National Institute of Materials Physics, Atomistilor 405bis, Magurele, Romania
[2] University of Warwick, Department of Physics, CV4 7AL Coventry, UK

S1. Capacitance measurements performed before the magnetocapacitance meaurements

After the temperature was set to 75K, the capacitance was continuously measured at zero DC applied voltage using an AC signal of 0.2 V amplitude and 1 kHz frequency. The purpose of this measurement was to estimate the contribution of the variations of the capacitance values induced by relaxations and other instabilities of the system. All other measurements were performed after 5 h from the moment the temperature of the system became stable and it was observed that the capacitance values varied with maximum 0.07% in 30 minutes. This variation of the capacitance values with time has a major impact on the C-V curves and consequently on the estimation of the MC values. In Figure S1 are displayed three consecutive C-V curves, obtained at 0 kOe applied magnetic field. It is worth noting that the shape of the first curve is different from the subsequent loops, especially at low voltage values. For DC voltage values lower than 0.5 V the difference between the capacitance values from the first and the second loop can be as high as 6% while for DC values close to 2.1 V this difference is about 0.05%. The second and the third loop have similar shapes and the difference between the capacitance values as low as 0.05% for all voltage values.

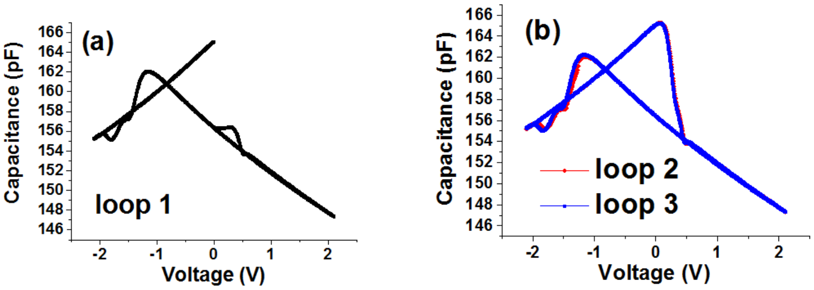

Figure S1 Three capacitance-voltage curves acquired consecutively at 0 kOe. The curves were represented separately for a better clarity, in (a) it is presented loop 1 which is the result of the first measurement and those from the second and the third are shown in (b)

S2. Capacitance-voltage measurements performed on PZT thin films with different thickness

The value of the dielectric permittivity of the PZT film (ε_{st}) was estimated from the capacitance-voltage measurements performed on three samples with PZT thickness of 25 nm, 50 nm and 100 nm prepared in the same conditions as the sample studied in this paper.

At 1 kHz, the experimental capacitance (C) of the LSMO-PZT-LSMO heterostructure is:

$$\frac{1}{C} = \frac{1}{C_i} + \frac{1}{C_p} = \frac{1}{C_i} - \frac{d_i}{\varepsilon_{st}\varepsilon_o A} + \frac{d}{\varepsilon_{st}\varepsilon_o A}$$

were ε_o is the dielectric permittivity of air C_i interface capacitance, d_i is the thickness of the interfaces, d is the total thickness of the heterostructure and A the area of the electrode. By representing $1/C$ vs. d for V_{DC} values where the spontaneous polarization is saturated, the dielectric permittivity of the PZT film was estimated to ~102 from the slope.

S3 Simulation of the experimental data

In Figure S3 it is represented the electrical circuit associated to the LSMO-PZT-LSMO heterostructure. The series circuit from the left side of Figure S3 is composed from the resistance R_L associated to the LSMO electrodes and two parallel RC circuits one corresponding to the interfaces (R_i and C_i) and the other one to the PZT film (R_p and C_p). This circuit is equivalent with a simple parallel RC circuit in which C and R is the total capacitance and resistance.

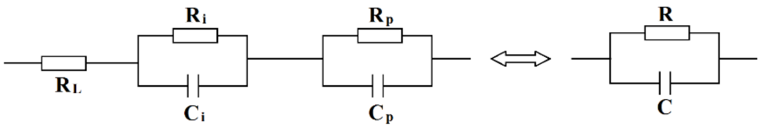

Figure S3 Equivalent circuit

The real and the imaginary parts of the impedance for these circuits are:

$$Z' = \frac{R}{1 + (\omega CR)^2}$$

$$Z" = \frac{\omega CR^2}{1 + (\omega CR)^2}$$

$$Z'_t = R_L + \frac{R_i}{1 + (\omega C_i R_i)^2} + \frac{R_P}{1 + (\omega C_P R_P)^2}$$

$$Z''_t = \frac{\omega C_i R_i^2}{1 + (\omega C_i R_i)^2} + \frac{\omega C_P R_P^2}{1 + (\omega C_P R_P)^2}$$

The general formula for the loss tangent is:

$$tg\delta = \frac{Z'}{Z"}$$

The total resistance R for the heterostructure is:

$$R = \frac{Z"}{\omega CZ'}$$

By inserting the above mathematical expression for R in to Z", the total capacitance C is:

$$C = \frac{1}{\omega Z"\left(1 + \left(\frac{Z'}{Z"}\right)^2\right)}$$

or:

$$C = \frac{1}{\omega Z"(1 + (tg\delta)^2)}$$

If $Z_t = Z$ then $Z'_t = Z'$ and $Z''_t = Z''$. In this case the total capacitance can be written as:

$$C = \frac{1}{\omega\, Z"_t(1 + (\, tg_t\delta\,)^2)}$$

where:

$$tg_t\delta = \frac{Z'_t}{Z"_t}$$

Considering that in this model it is assumed that the only magnetic field dependent component of the circuit is R_L, the magnetocapacitance is:

$$MC = \left[\frac{1 + tg_t^2\delta(0)}{1 + tg_t^2\delta(H)} - 1\right]100$$

The magnetolosses are thus given by:

$$Mtg\delta = \left(\frac{Z'_t(H) - Z'_t(0)}{Z'_t(0)}\right)100 = \left(\frac{R_L(H) - R_L(0)}{R_L(0) + \frac{R_i}{1 + (\omega C_i R_i)^2} + \frac{R_P}{1 + (\omega C_P R_P)^2}}\right)100$$

or:

$$Mtg\delta = m[R_L(H) - R_L(0)]100$$

where

$$m = \frac{1}{R_L(0) + \frac{R_i}{1 + (\omega C_i R_i)^2} + \frac{R_P}{1 + (\omega C_P R_P)^2}}$$
